# Evaluating severity of white matter lesions from computed tomography images with convolutional neural network

**DOI:** 10.1007/s00234-020-02410-2

**Published:** 2020-04-13

**Authors:** Johanna Pitkänen, Juha Koikkalainen, Tuomas Nieminen, Ivan Marinkovic, Sami Curtze, Gerli Sibolt, Hanna Jokinen, Daniel Rueckert, Frederik Barkhof, Reinhold Schmidt, Leonardo Pantoni, Philip Scheltens, Lars-Olof Wahlund, Antti Korvenoja, Jyrki Lötjönen, Timo Erkinjuntti, Susanna Melkas

**Affiliations:** 1 Department of Neurology, University of Helsinki and Helsinki University Hospital, PO Box 302, 00029 HUS Helsinki, Finland; 2 Combinostics Ltd., Tampere, Finland and VTT Technical Research Centre of Finland, Tampere, Finland; 3 Department of Psychology and Logopedics, Faculty of Medicine, University of Helsinki, Helsinki, Finland; 4 Biomedical Image Analysis Group, Department of Computing, Imperial College London, London, UK; 5 Department of Radiology and Nuclear Medicine, Neuroscience Campus Amsterdam, VU University Medical Center, Amsterdam, The Netherlands; 6 Institutes of Neurology and Healthcare Engineering, University College London, London, UK; 7 NIHR Biomedical Research Centre at University College London Hospitals NHS Foundation Trust and University College London, London, England UK; 8 Department of Neurology, Neuroscience Campus Amsterdam, VU University Medical Center, Amsterdam, Netherlands; 9 L. Sacco Department of Biomedical and Clinical Sciences, University of Milan, Milan, Italy; 10 Alzheimer Center and Department of Neurology, Neuroscience Campus Amsterdam, VU University Medical Center, Amsterdam, Netherlands; 11 Department of Neurobiology, Care Sciences and Society, Division of Clinical Geriatrics, Karolinska Institutet, Stockholm, Sweden; 12 HUS Medical Imaging Center, Radiology, University of Helsinki and Helsinki University Hospital, Helsinki, Finland

**Keywords:** Cerebral small vessel disease, Convolutional neural network, Computed tomography, Machine learning, White matter lesions

## Abstract

**Purpose:**

Severity of white matter lesion (WML) is typically evaluated on magnetic resonance images (MRI), yet the more accessible, faster, and less expensive method is computed tomography (CT). Our objective was to study whether WML can be automatically segmented from CT images using a convolutional neural network (CNN). The second aim was to compare CT segmentation with MRI segmentation.

**Methods:**

The brain images from the Helsinki University Hospital clinical image archive were systematically screened to make CT-MRI image pairs. Selection criteria for the study were that both CT and MRI images were acquired within 6 weeks. In total, 147 image pairs were included. We used CNN to segment WML from CT images. Training and testing of CNN for CT was performed using 10-fold cross-validation, and the segmentation results were compared with the corresponding segmentations from MRI.

**Results:**

A Pearson correlation of 0.94 was obtained between the automatic WML volumes of MRI and CT segmentations. The average Dice similarity index validating the overlap between CT and FLAIR segmentations was 0.68 for the Fazekas 3 group.

**Conclusion:**

CNN-based segmentation of CT images may provide a means to evaluate the severity of WML and establish a link between CT WML patterns and the current standard MRI-based visual rating scale.

## Introduction

White matter lesions (WML) are a surrogate for cerebral small vessel disease (SVD), which is the major cause of accumulating vascular burden in aging populations. Severe WML in stroke patients are associated with a risk of complications after thrombolysis [1] and poor prognosis after carotid endarterectomy [2]. Other well-documented consequences of severe WML are cognitive impairment, gait disturbances, depression, urine incontinence, and the eventual loss of independence and risk for permanent institutionalization [3, 4].

The most common method for grading WML extent has been the Fazekas visual rating scale developed for MRI [5, 6]. It was preceded by several proposals for CT-based visual rating scales by the authors Gorter [7], Blennow et al. [8], van Swieten et al. [9], and Wahlund et al. [10] which have not been widely adopted in clinical practice [6, 11].

Computer-aided image analysis and machine learning methods are increasingly used in medicine. They enable automated and quantitative analyses of large image databases and help to develop tools that complement the manual visual assessment. Advances in machine learning, especially in the field of deep learning, have improved the ability to identify, quantify, and classify patterns in medical images [11].

Deep learning methods, in particular convolutional neural networks (CNNs), have become the state-of-the-art methods for medical image analysis tasks. Modern central processing units (CPUs) and graphics processing units (GPUs) are powerful enough to process large amount of data with advanced learning algorithms [12]. CNNs take a large number of training samples as an input and build a model with a vast number of parameters that will predict the output based on the training examples. CNNs use convolution operation to learn the features such as edges, patterns, and colors from the input images [13]. They have been applied in several image processing tasks such as image segmentation [14] and image classification [15]. Recently, CNNs have also been applied to medical image analysis [16, 17].

In this study, the objective was to study if the WML can be automatically segmented from CT images using CNN. The aim was also to compare CT segmentation with MRI segmentation.

## Methods

### Participants and design

Brain images from the Helsinki University Hospital clinical image archive were systemically screened by qualified healthcare professionals from January 2014 to December 2016 to make CT-MRI image pairs. The images were from the Helsinki University Hospital, and from five area hospitals in the Helsinki region. MRIs were acquired with Siemens and Philips scanners, and CT scanners included Siemens and GE devices.

Thirteen FLAIR images were sagittal 3D images with in-plane resolution 0.45–0.47 mm and slice thickness 0.9–1.2 mm. The remaining 136 images were 2D axial images with in-plane resolution 0.43–0.98 mm and slice thickness 4.0–5.0 mm. The in-plane resolution of CT images was 0.41–1.0 mm, and the slice thickness was 1.0–5.0 mm.

Selection criteria for the study were that both CT and MRI images were acquired and the time interval between CT and MRI imaging was less than 6 weeks. Images with tumors, cortical infarcts, hematomas (except microbleeds), and multiple sclerosis lesions and contusions were excluded. The images were divided into three Fazekas groups (Fazekas 0–1 = no to mild WML, Fazekas 2 = moderate WML, Fazekas 3 = severe WML) according to radiologists’ evaluation of the MRI image. The evaluation was made both by general radiologists and neuroradiologists. In total, 147 image pairs were included in the study (Table 1).

**Table 1:** Demographics of the dataset

	Mean age	SD age	% females
All *N* = 147	71.2	9.7	55%
Fazekas 0–1 *N* = 50	65.7	11.5	58%
Fazekas 2 *N* = 48	73.4	7.1	56%
Fazekas 3 *N* = 49	74.7	7.2	51%

Ethical review for retrospective analysis of imaging data collected prospectively as part of routine clinical care is not required at our institution. The analysis of image pairs was anonymized and no clinical data was handled in connection to this analysis.

### Automated image analysis

**Fig. 1:**
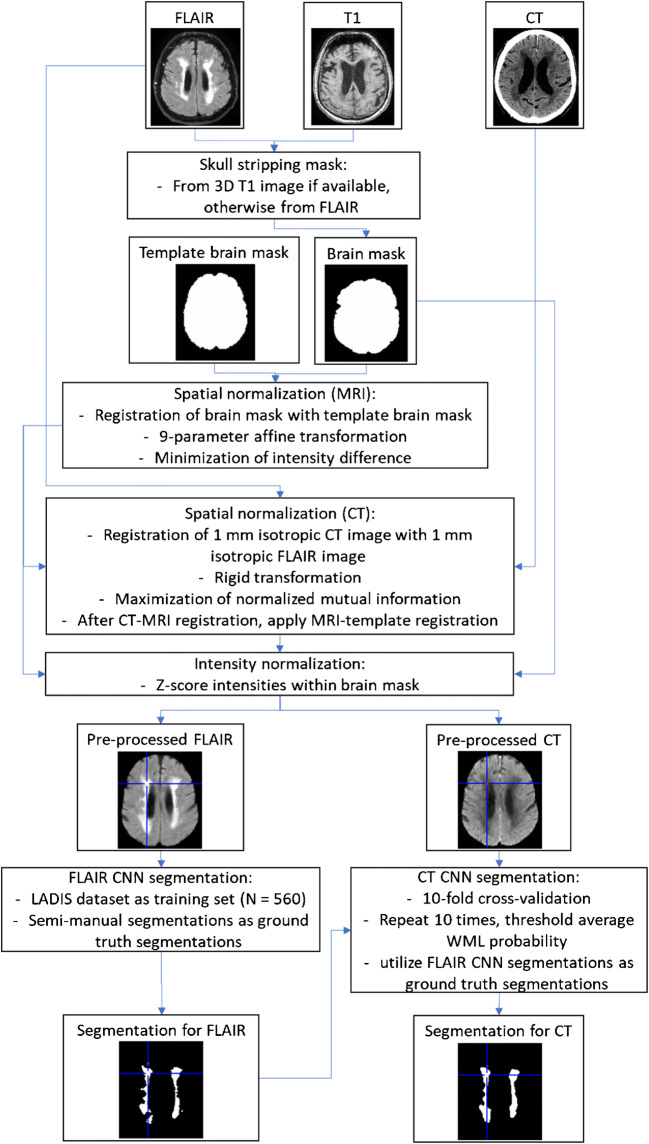
Flowchart of the analysis (*n* = 147)

The analysis pipeline is presented in Fig. 1. The pre-processing steps included skull-stripping, coarse spatial normalization, and coarse intensity normalization of the images. The skull-stripping (brain extraction) of the MRI FLAIR images was performed using the cNeuro® cMRI image quantification tool (Combinostics Ltd., Tampere, Finland). Spatial normalization was performed by registering the binary brain mask to the corresponding brain mask of a mean anatomical template image using 9-degree of freedom affine registration. A CT image was registered with the FLAIR image using rigid registration by maximizing the normalized mutual information. Finally, the intensities were normalized by *z*-scoring within the brain mask.

The resulting pre-processed images were used as inputs in CNN segmentation. Two CNN models were created. (1) CNN for MRI was developed using FLAIR segmentations from the LADIS study (Leukoaraiosis and Disability study) as training data (560 FLAIR images with semi-manually segmented WML). (2) CNN for CT was developed using MRI segmentations from the MRI-CT pairs as training data.

MRI and CT images were segmented using CNN without and with 10-fold cross-validation, respectively. The CT images were selected randomly so that 90% of the cases established the training set, and the remaining 10% of the cases established the test set. This was repeated ten times so that each case was once used in a test set. The WML segmentations of the FLAIR images were used as the ground truth segmentations for training. To improve the robustness of the segmentation, the 10-fold cross-validation was repeated ten times so that ten separate segmentations were obtained for each CT image. The CNN segmentation gives the probability of the WML as an output. The final segmentation was generated by averaging the probabilities of the ten segmentations, and thresholding the average probability using a value of 0.25.

CNN segmentations (both FLAIR and CT) were performed using U-shaped CNN called uResNet [15]. In this study, we used the network architecture proposed by Guerrero et al. [18] that was originally developed for the segmentation of white matter hyperintensities and stroke lesions from FLAIR images. This network, without any further modifications, was implemented using Theano 0.9.0 (http://www.deeplearning.net/software/theano/) deep learning Python (Python 2.7) library. The CNN was trained using large image patches (64 × 64). This allows the network to learn the high- and low-level features from the input images. During the training, CNN parameters were optimized so that the error between the predicted segmentations and “ground truth” segmentations was as small as possible.

### Statistical analysis

The accuracy of the CT WML segmentations was validated by comparing the segmentations to the corresponding segmentations from the FLAIR images. The accuracy of the CT segmentations was evaluated by keeping the segmentation of FLAIR images as a ground truth. The Dice overlap measures that the ratio of voxels segmented as WML in both images and the voxels segmented as WML in CT and in FLAIR: $\mathrm{Dice}=\frac{2\left|X\cap Y\right|}{\left|X\right|+\left|Y\right|}$, where |*X*| and |*Y*| are the WML volumes of the CT and FLAIR segmentations, and |*X* ∩ *Y*| is the volume of voxels segmented as WML in both CT and FLAIR. In addition, the accuracy of the segmentation was evaluated by studying the volume of correctly and incorrectly segmented voxels. The correlation of the volumes of CT and FLAIR segmentations was evaluated by computing the Pearson correlation. In addition, the Fazekas score was estimated from the WML volumes by searching the optimal thresholds for the three Fazekas groups used (0–1, 2, and 3). These computations were performed using 10-fold cross-validation.

## Results

**Fig. 2:**
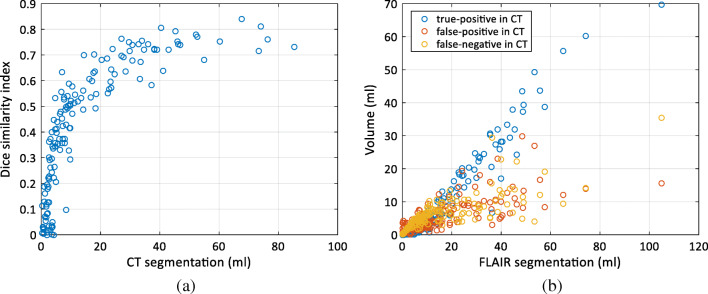
The accuracy of the segmentation of CT images. **a** The Dice similarity index as a function of the WML volume. The distribution of the WML volumes as a function of Fazekas score. **b** The volumes of correctly and incorrectly segmented voxels in CT images as compared with the segmentation of FLAIR images

The Dice similarity index validating the overlap between CT and FLAIR segmentations is presented as the function of the WML volume in Fig. 2a. As expected, the index values are low for small WML volumes: the average Dice similarity index was 0.43 for the whole dataset. However, the more WML there are, the higher values are obtained: the average Dice similarity index value for the Fazekas 3 group was 0.68. The volumes of correctly segmented voxels, the voxels segmented as WML in CT but as background in FLAIR, and the voxels segmented as WML in FLAIR but as background in CT are presented in Fig. 2b.

**Fig. 3:**
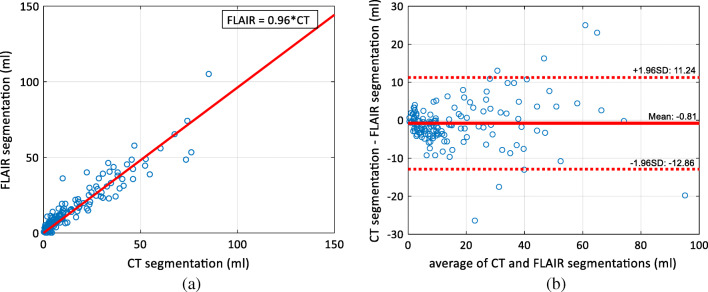
The correlation of the volumes. **a** The correlation between the WML volumes segmented from CT and FLAIR images. The correlation coefficient was 0.94. **b** The Bland-Altman plot for the differences of CT and FLAIR segmentations

The correlation of the WML volumes of the CT and FLAIR segmentations is shown in Fig. 3a. The volumes of the CT and FLAIR segmentations are strongly correlated (correlation coefficient 0.94). Also, the slope of the curve fitted to the data is 0.96, close to 1—i.e., the CT segmentation neither underestimates nor overestimates the WML volume as compared with the FLAIR segmentation, which can be seen also in the Bland-Altman plot in Fig. 3b.

**Fig. 4:**
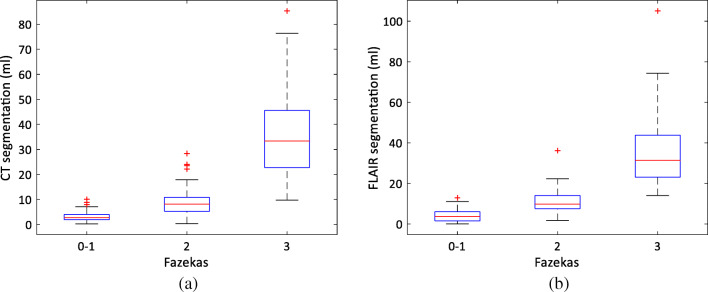
The distribution of the WML volumes as a function of Fazekas score **a** for CT and **b** for FLAIR segmentations

The distributions of the WML volumes for different Fazekas groups for CT and FLAIR segmentations are shown in Fig. 4 a and b, respectively. The Fazekas groups have clearly distinct distributions in both cases, and qualitatively, the CT and FLAIR distributions are very similar. Table 2 presents the results for estimating the Fazekas scores from the WML volumes when compared with the ground truth visual ratings. The score was correctly estimated in 78% of cases from both CT and FLAIR images.

**Table 2:** Confusion matrix of the estimated Fazekas scores based on the automatic WML volumes using CT (share of correct estimates = 0.78) and FLAIR (share of correct estimates = 0.78)

CT		Automatic score		
		0–1	2	3
Visual score	0–1	43	7	0
	2	12	28	8
	3	0	5	44
FLAIR		Automatic score		
		0–1	2	3
Visual score	0–1	37	13	0
	2	7	37	4
	3	0	9	40

**Fig. 5:**
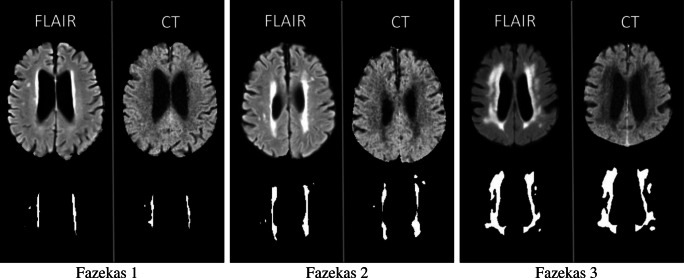
Examples of the FLAIR and CT WML segmentations for the three Fazekas groups

Figure *5* shows the example segmentations for the CT and FLAIR images of patients from each Fazekas group. This shows that especially when the WML volume is high, the CT segmentation is able to produce corresponding results with the FLAIR segmentation.

## Discussion

Our study suggests that the severity of WML can be estimated from CT images, using automated image analysis methods, with results very similar to those using the Fazekas scale for MRI images. These methods provide means for volumetric assessment of the burden of WML as an alternative to visual scaling. There was a high correlation of 0.94 obtained between the automatic WML volumes of MRI and CT segmentations. The ratings produced automatically both from CT and MRI were equal to visual ratings in 78% of the cases.

Previously, an automated method for quantifying CT cerebral WML has been under evaluation in a multicenter validation study in the UK [19]. The automated WML volume correlation at MR imaging was 0.85 and at CT imaging 0.71 when compared with expert-delineated WML volumes. The study sample in UK was acute ischemic stroke cases.

In general, the constraint of utilizing CT instead of MRI is the impaired detection of small lesions including punctate and early confluent changes. However, CT seems to be sufficient when using a multi-detector CT with coronal and sagittal reformats [20]. CT is also often used in dementia imaging in clinical practice [21]. Among acute stroke patients, non-contrast CT is the most common initial imaging modality in clinical practice [22]. From the clinical point of view, detecting moderate and severe WML is more relevant than detecting early phase WML (pre-mild or mild), because acute clinical complications and risks are associated with moderate and severe WML [1, 4, 23]. Early phase WML is more relevant in younger age groups participating in follow-up and in intervention studies. In this setting, the patients are more likely to undergo MRI.

We used the automated FLAIR WML segmentations from the LADIS cohort as the ground truth segmentations when training the CNN model and validating the CT segmentation results. Our previous cross-validated study [24] has shown that the CNN-based WML segmentation on MRIs produces very similar results when compared with the semi-manual segmentation (correlation 0.99, average Dice similarity index 0.72). This suggests that the MRI-based CNN WML segmentation can be used as ground truth in training CT CNN models and can also be used in validation.

A relatively small dataset is a limitation in this study, and a totally independent validation set is needed in future studies. It is possible that our findings are in some extent obscured by the presence of lacunes, but probably this influence is of minor importance because lacunes have a distinct morphology that does not confuse with WML. Microbleeds were not regarded because they are invisible on CT. In the present study, patients with concomitant lesions like cortical infarcts or tumor edema were excluded, which is a limitation. In future studies, such combined lesions could be evaluated with deep learning requiring a larger training set with good representation of different lesion types. Also, the lack of clinical data is a limitation in our study.

The strength of our study is that the images were unselected. The patients were not exclusively stroke patients nor other neurological patients. The CT and MRI equipment as well as the imaging parameters varied. Therefore, the results and the models are more likely to generalize to other datasets. While this will likely increase variability in segmentation results, we consider that the scanner differences and different scan parameters (such as different kV or double energy) do not affect the results as far as the analysis is restricted to moderate or severe WML. The influence of different variables on variance could be analyzed in future studies.

Automated volumetric rating could direct radiologists towards a uniform evaluation of WML and might increase clinician’s alertness for WML and its influences on treatment and outcomes. Automated rating enables a variety of analyses in cohorts of stroke patients and other neurological patients and studies can be cross-evaluated worldwide. In the future, it will be interesting to study the correlation with clinical data to see if CT segmentation leads to similar results in terms of clinical correlation when compared with MRI segmentation. Although the present study suggests a clinical solution, the method is still not all-inclusive and thus calls for further research, for example, for segmentation in Fazekas grades 1 and 2. More uniform imaging parameters will likely aid in achieving this goal.
